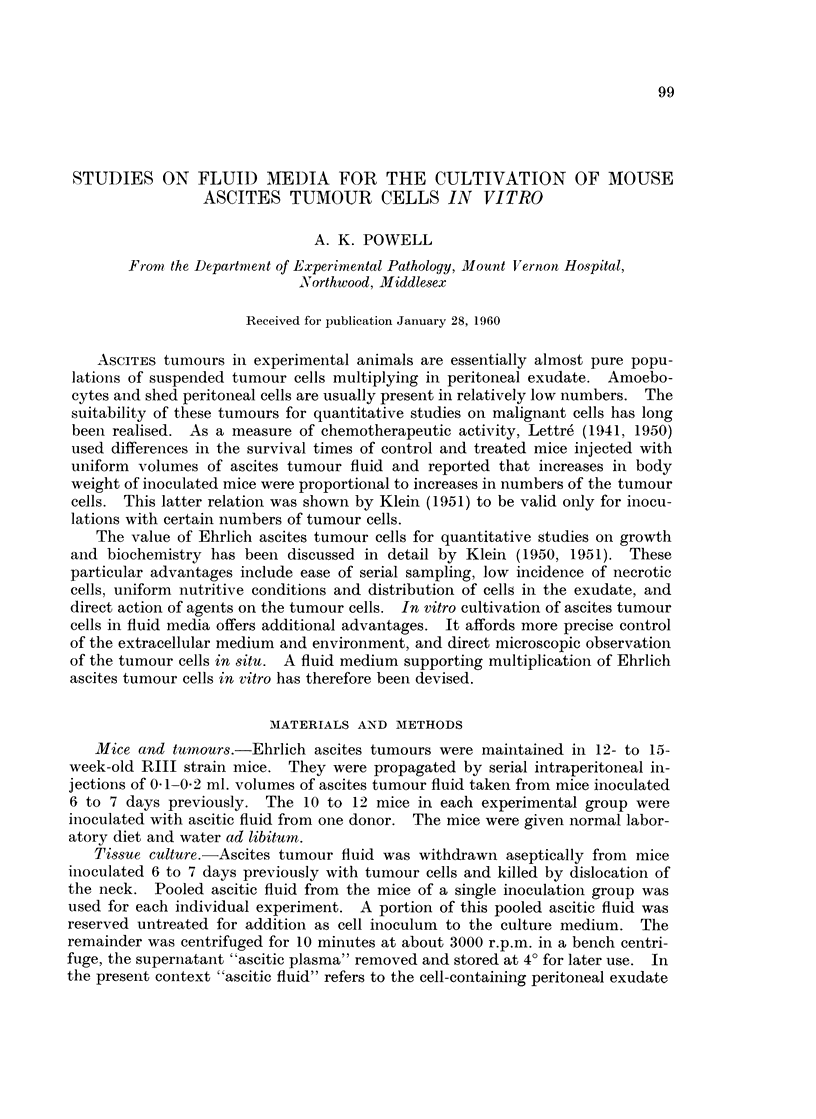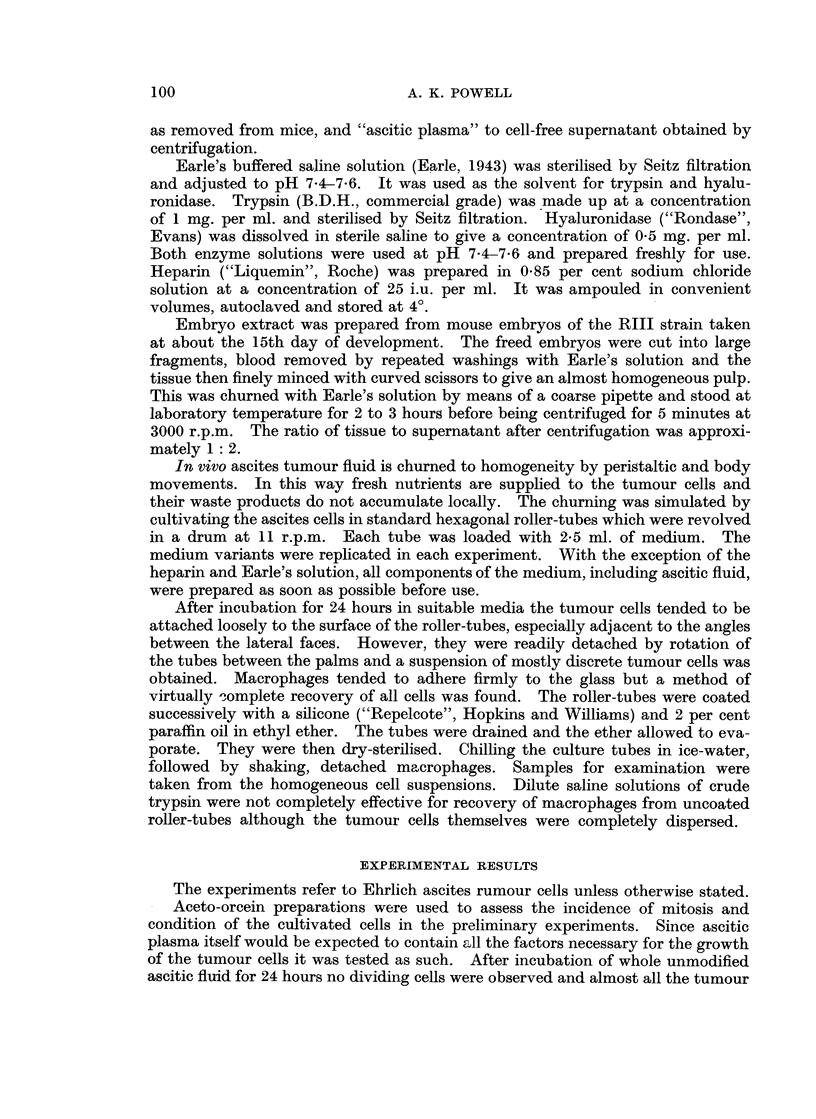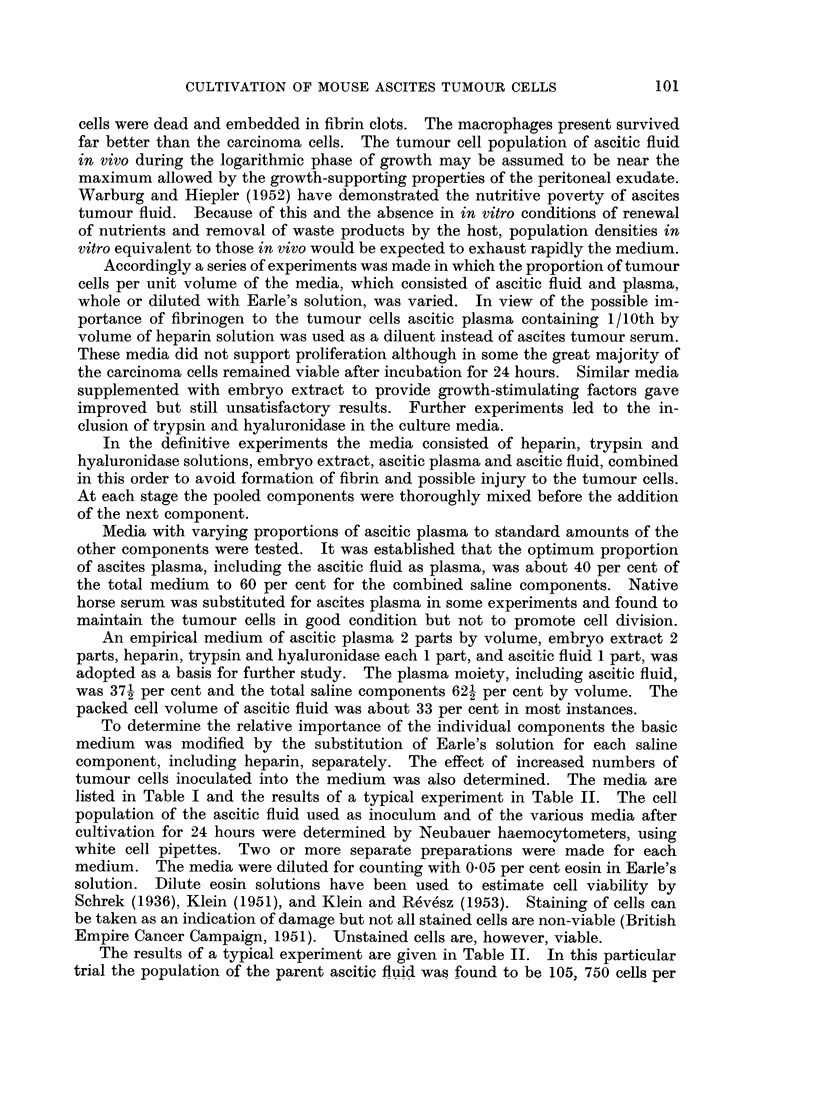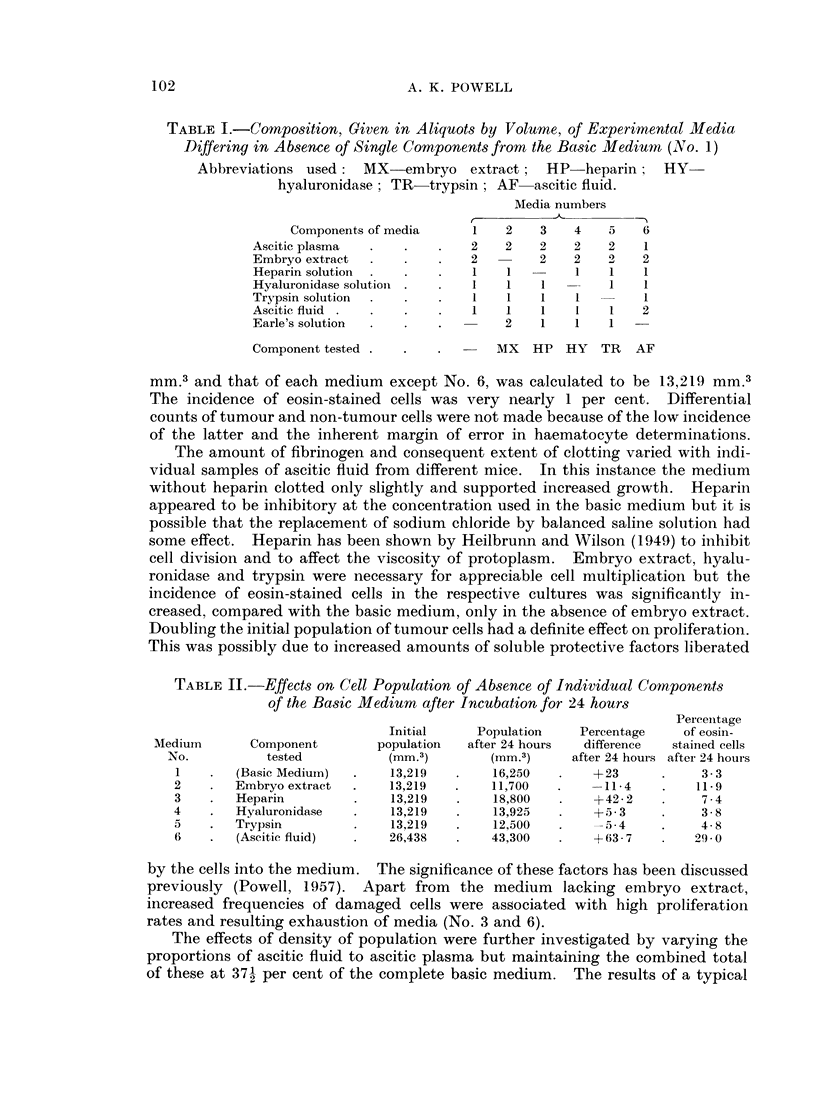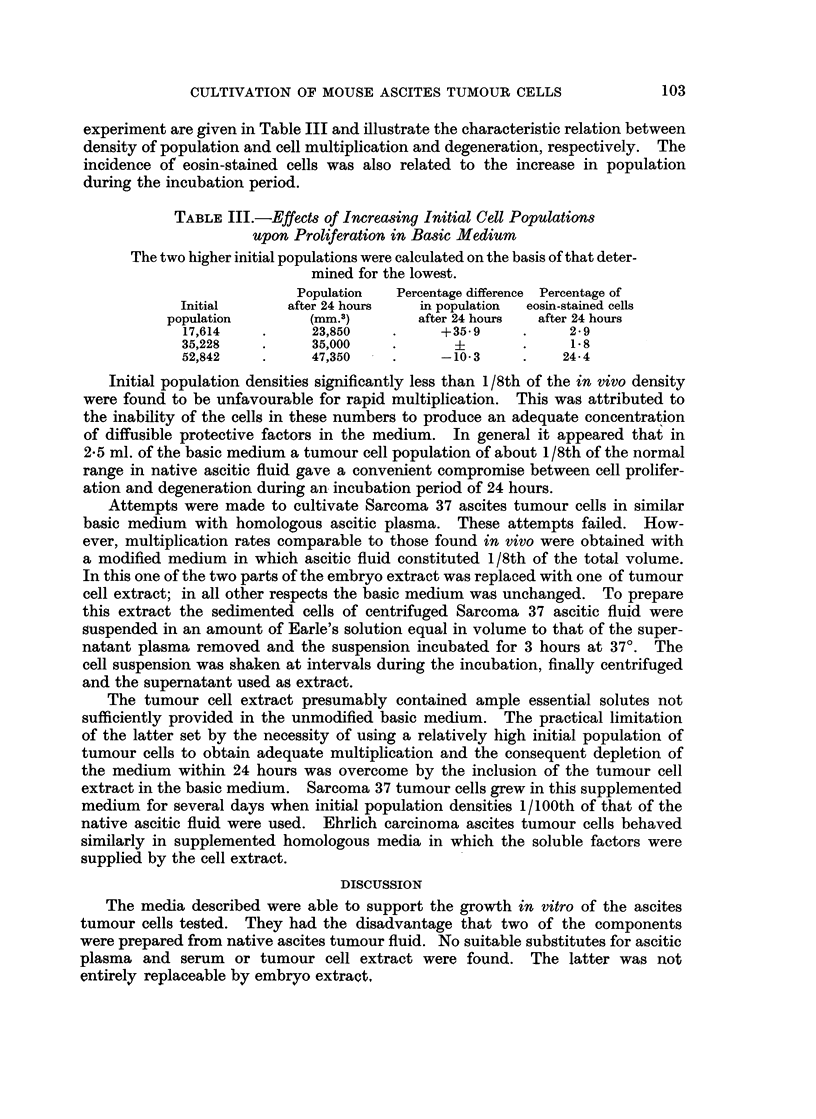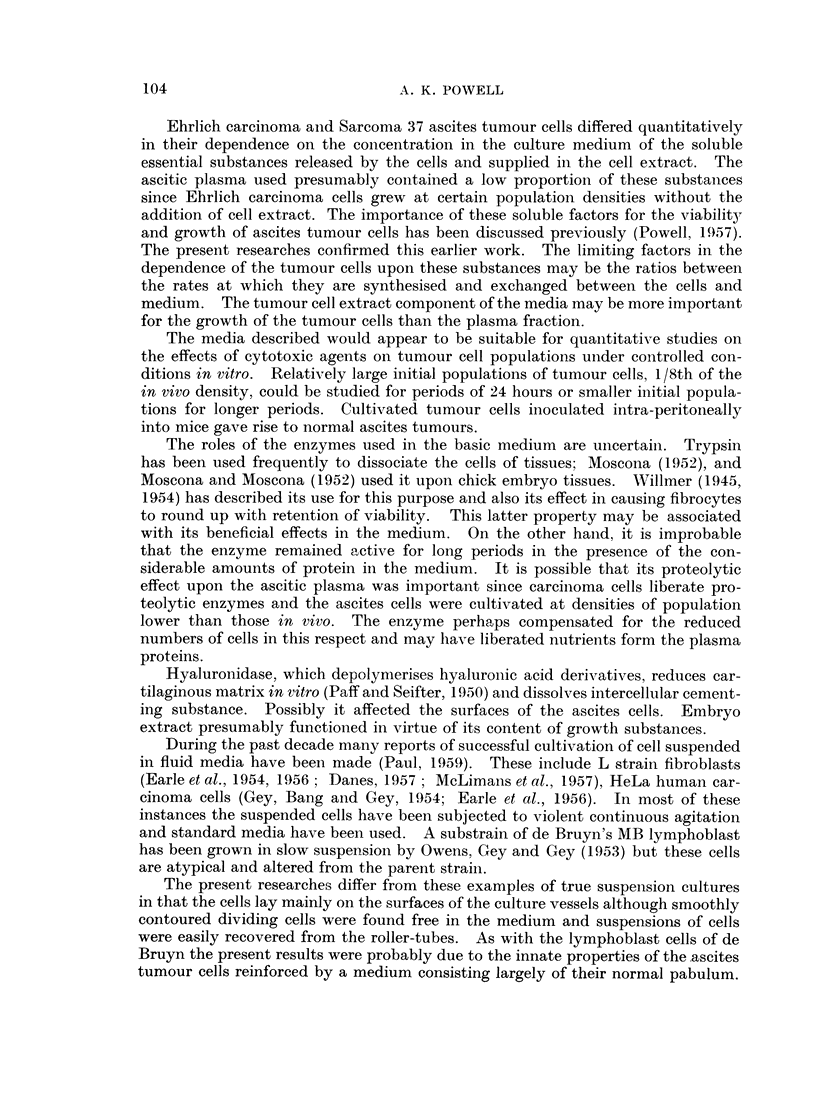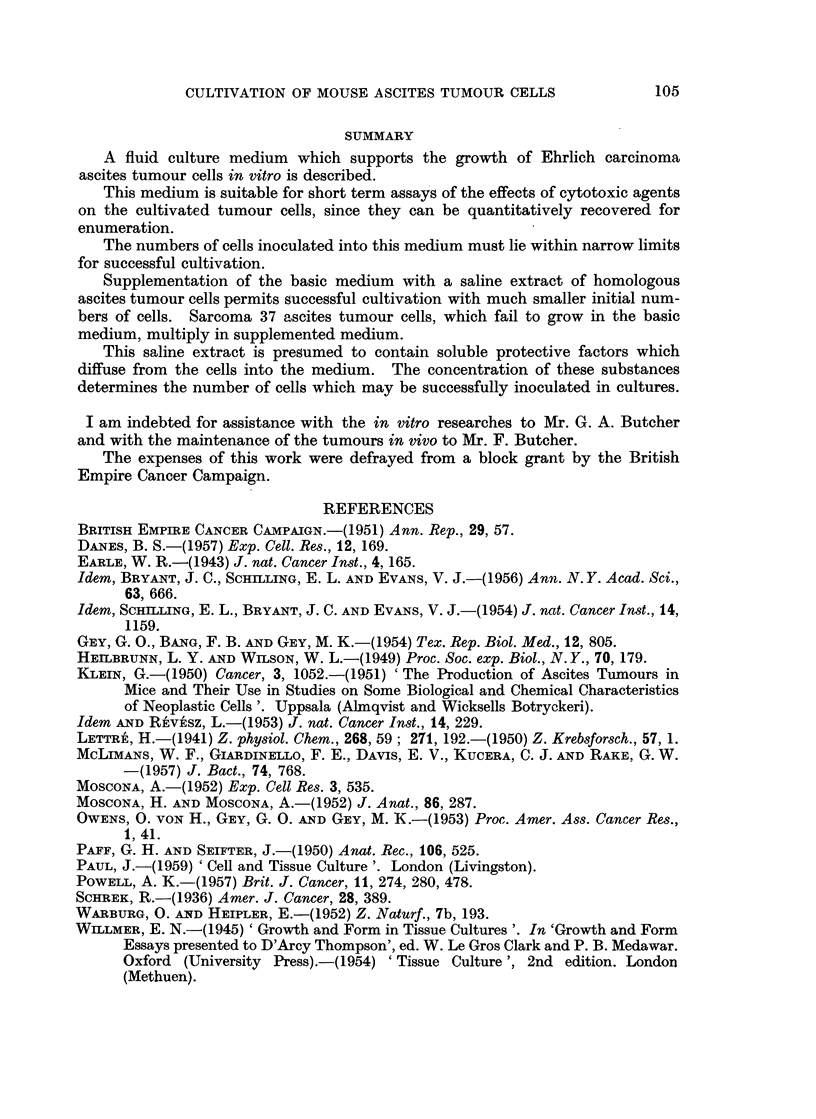# Studies on Fluid Media for the Cultivation of Mouse Ascites Tumour Cells In Vitro

**DOI:** 10.1038/bjc.1960.12

**Published:** 1960-03

**Authors:** A. K. Powell


### STUDIES ON FLUID MEDIA FOR THE CULTIVATION OF MOUSE ASCITES TUMOUR CELLS *IN VITRO*

A. K. POWELL

*From the Department of Experimental Pathology, Mount Vernon Hospital, Northwood, Middlesex*

Received for publication January 28, 1960

ASCITES tumours in experimental animals are essentially almost pure populations of suspended tumour cells multiplying in peritoneal exudate. Amoebocytes and shed peritoneal cells are usually present in relatively low numbers. The suitability of these tumours for quantitative studies on malignant cells has long been realised. As a measure of chemotherapeutic activity, Lettré (1941, 1950) used differences in the survival times of control and treated mice injected with uniform volumes of ascites tumour fluid and reported that increases in body weight of inoculated mice were proportional to increases in numbers of the tumour cells. This latter relation was shown by Klein (1951) to be valid only for inoculations with certain numbers of tumour cells.

The value of Ehrlich ascites tumour cells for quantitative studies on growth and biochemistry has been discussed in detail by Klein (1950, 1951). These particular advantages include ease of serial sampling, low incidence of necrotic cells, uniform nutritive conditions and distribution of cells in the exudate, and direct action of agents on the tumour cells. *In vitro* cultivation of ascites tumour cells in fluid media offers additional advantages. It affords more precise control of the extracellular medium and environment, and direct microscopic observation of the tumour cells *in situ*. A fluid medium supporting multiplication of Ehrlich ascites tumour cells *in vitro* has therefore been devised.

### MATERIALS AND METHODS

*Mice and tumours.*—Ehrlich ascites tumours were maintained in 12- to 15-week-old RIII strain mice. They were propagated by serial intraperitoneal injections of 0·1–0·2 ml. volumes of ascites tumour fluid taken from mice inoculated 6 to 7 days previously. The 10 to 12 mice in each experimental group were inoculated with ascitic fluid from one donor. The mice were given normal laboratory diet and water *ad libitum.*

*Tissue culture.*—Ascites tumour fluid was withdrawn aseptically from mice inoculated 6 to 7 days previously with tumour cells and killed by dislocation of the neck. Pooled ascitic fluid from the mice of a single inoculation group was used for each individual experiment. A portion of this pooled ascitic fluid was reserved untreated for addition as cell inoculum to the culture medium. The remainder was centrifuged for 10 minutes at about 3000 r.p.m. in a bench centrifuge, the supernatant "ascitic plasma" removed and stored at 4° for later use. In the present context "ascitic fluid" refers to the cell-containing peritoneal exudate as removed from mice, and "ascitic plasma" to cell-free supernatant obtained by centrifugation.

Earle's buffered saline solution (Earle, 1943) was sterilised by Seitz filtration and adjusted to pH 7·4–7·6. It was used as the solvent for trypsin and hyaluronidase. Trypsin (B.D.H., commercial grade) was made up at a concentration of 1 mg. per ml. and sterilised by Seitz filtration. Hyaluronidase ("Rondase", Evans) was dissolved in sterile saline to give a concentration of 0·5 mg. per ml. Both enzyme solutions were used at pH 7·4–7·6 and prepared freshly for use. Heparin ("Liquemin", Roche) was prepared in 0·85 per cent sodium chloride solution at a concentration of 25 i.u. per ml. It was ampouled in convenient volumes, autoclaved and stored at 4°.

Embryo extract was prepared from mouse embryos of the RIII strain taken at about the 15th day of development. The freed embryos were cut into large fragments, blood removed by repeated washings with Earle's solution and the tissue then finely minced with curved scissors to give an almost homogeneous pulp. This was churned with Earle's solution by means of a coarse pipette and stood at laboratory temperature for 2 to 3 hours before being centrifuged for 5 minutes at 3000 r.p.m. The ratio of tissue to supernatant after centrifugation was approximately 1 : 2.

*In vivo* ascites tumour fluid is churned to homogeneity by peristaltic and body movements. In this way fresh nutrients are supplied to the tumour cells and their waste products do not accumulate locally. The churning was simulated by cultivating the ascites cells in standard hexagonal roller-tubes which were revolved in a drum at 11 r.p.m. Each tube was loaded with 2·5 ml. of medium. The medium variants were replicated in each experiment. With the exception of the heparin and Earle's solution, all components of the medium, including ascitic fluid, were prepared as soon as possible before use.

After incubation for 24 hours in suitable media the tumour cells tended to be attached loosely to the surface of the roller-tubes, especially adjacent to the angles between the lateral faces. However, they were readily detached by rotation of the tubes between the palms and a suspension of mostly discrete tumour cells was obtained. Macrophages tended to adhere firmly to the glass but a method of virtually complete recovery of all cells was found. The roller-tubes were coated successively with a silicone ("Repelcote", Hopkins and Williams) and 2 per cent paraffin oil in ethyl ether. The tubes were drained and the ether allowed to evaporate. They were then dry-sterilised. Chilling the culture tubes in ice-water, followed by shaking, detached macrophages. Samples for examination were taken from the homogeneous cell suspensions. Dilute saline solutions of crude trypsin were not completely effective for recovery of macrophages from uncoated roller-tubes although the tumour cells themselves were completely dispersed.

### EXPERIMENTAL RESULTS

The experiments refer to Ehrlich ascites rumour cells unless otherwise stated.

Aceto-orcein preparations were used to assess the incidence of mitosis and condition of the cultivated cells in the preliminary experiments. Since ascitic plasma itself would be expected to contain all the factors necessary for the growth of the tumour cells it was tested as such. After incubation of whole unmodified ascitic fluid for 24 hours no dividing cells were observed and almost all the tumour cells were dead and embedded in fibrin clots. The macrophages present survived far better than the carcinoma cells. The tumour cell population of ascitic fluid *in vivo* during the logarithmic phase of growth may be assumed to be near the maximum allowed by the growth-supporting properties of the peritoneal exudate. Warburg and Hiepler (1952) have demonstrated the nutritive poverty of ascites tumour fluid. Because of this and the absence in *in vitro* conditions of renewal of nutrients and removal of waste products by the host, population densities *in vitro* equivalent to those *in vivo* would be expected to exhaust rapidly the medium.

Accordingly a series of experiments was made in which the proportion of tumour cells per unit volume of the media, which consisted of ascitic fluid and plasma, whole or diluted with Earle's solution, was varied. In view of the possible importance of fibrinogen to the tumour cells ascitic plasma containing 1/10th by volume of heparin solution was used as a diluent instead of ascites tumour serum. These media did not support proliferation although in some the great majority of the carcinoma cells remained viable after incubation for 24 hours. Similar media supplemented with embryo extract to provide growth-stimulating factors gave improved but still unsatisfactory results. Further experiments led to the inclusion of trypsin and hyaluronidase in the culture media.

In the definitive experiments the media consisted of heparin, trypsin and hyaluronidase solutions, embryo extract, ascitic plasma and ascitic fluid, combined in this order to avoid formation of fibrin and possible injury to the tumour cells. At each stage the pooled components were thoroughly mixed before the addition of the next component.

Media with varying proportions of ascitic plasma to standard amounts of the other components were tested. It was established that the optimum proportion of ascites plasma, including the ascitic fluid as plasma, was about 40 per cent of the total medium to 60 per cent for the combined saline components. Native horse serum was substituted for ascites plasma in some experiments and found to maintain the tumour cells in good condition but not to promote cell division.

An empirical medium of ascitic plasma 2 parts by volume, embryo extract 2 parts, heparin, trypsin and hyaluronidase each 1 part, and ascitic fluid 1 part, was adopted as a basis for further study. The plasma moiety, including ascitic fluid, was $37\frac{1}{2}$ per cent and the total saline components $62\frac{1}{2}$ per cent by volume. The packed cell volume of ascitic fluid was about 33 per cent in most instances.

To determine the relative importance of the individual components the basic medium was modified by the substitution of Earle's solution for each saline component, including heparin, separately. The effect of increased numbers of tumour cells inoculated into the medium was also determined. The media are listed in Table I and the results of a typical experiment in Table II. The cell population of the ascitic fluid used as inoculum and of the various media after cultivation for 24 hours were determined by Neubauer haemocytometers, using white cell pipettes. Two or more separate preparations were made for each medium. The media were diluted for counting with 0·05 per cent eosin in Earle's solution. Dilute eosin solutions have been used to estimate cell viability by Schrek (1936), Klein (1951), and Klein and Révész (1953). Staining of cells can be taken as an indication of damage but not all stained cells are non-viable (British Empire Cancer Campaign, 1951). Unstained cells are, however, viable.

The results of a typical experiment are given in Table II. In this particular trial the population of the parent ascitic fluid was found to be 105, 750 cells per

TABLE I.—*Composition, Given in Aliquots by Volume, of Experimental Media Differing in Absence of Single Components from the Basic Medium* (*No.* 1)

Abbreviations used: MX—embryo extract; HP—heparin; HY—hyaluronidase; TR—trypsin; AF—ascitic fluid.

| Components of media | Media numbers 1 | 2 | 3 | 4 | 5 | 6 |
|---|---|---|---|---|---|---|
| Ascitic plasma | 2 | 2 | 2 | 2 | 2 | 1 |
| Embryo extract | 2 | — | 2 | 2 | 2 | 2 |
| Heparin solution | 1 | 1 | — | 1 | 1 | 1 |
| Hyaluronidase solution | 1 | 1 | 1 | — | 1 | 1 |
| Trypsin solution | 1 | 1 | 1 | 1 | — | 1 |
| Ascitic fluid | 1 | 1 | 1 | 1 | 1 | 2 |
| Earle's solution | — | 2 | 1 | 1 | 1 | — |
| Component tested | — | MX | HP | HY | TR | AF |

mm.$^3$ and that of each medium except No. 6, was calculated to be 13,219 mm.$^3$ The incidence of eosin-stained cells was very nearly 1 per cent. Differential counts of tumour and non-tumour cells were not made because of the low incidence of the latter and the inherent margin of error in haematocyte determinations.

The amount of fibrinogen and consequent extent of clotting varied with individual samples of ascitic fluid from different mice. In this instance the medium without heparin clotted only slightly and supported increased growth. Heparin appeared to be inhibitory at the concentration used in the basic medium but it is possible that the replacement of sodium chloride by balanced saline solution had some effect. Heparin has been shown by Heilbrunn and Wilson (1949) to inhibit cell division and to affect the viscosity of protoplasm. Embryo extract, hyaluronidase and trypsin were necessary for appreciable cell multiplication but the incidence of eosin-stained cells in the respective cultures was significantly increased, compared with the basic medium, only in the absence of embryo extract. Doubling the initial population of tumour cells had a definite effect on proliferation. This was possibly due to increased amounts of soluble protective factors liberated

TABLE II.—*Effects on Cell Population of Absence of Individual Components of the Basic Medium after Incubation for* 24 *hours*

| Medium No. | Component tested | Initial population (mm.$^3$) | Population after 24 hours (mm.$^3$) | Percentage difference after 24 hours | Percentage of eosin-stained cells after 24 hours |
|---|---|---|---|---|---|
| 1 | (Basic Medium) | 13,219 | 16,250 | +23 | 3·3 |
| 2 | Embryo extract | 13,219 | 11,700 | −11·4 | 11·9 |
| 3 | Heparin | 13,219 | 18,800 | +42·2 | 7·4 |
| 4 | Hyaluronidase | 13,219 | 13,925 | +5·3 | 3·8 |
| 5 | Trypsin | 13,219 | 12,500 | −5·4 | 4·8 |
| 6 | (Ascitic fluid) | 26,438 | 43,300 | +63·7 | 29·0 |

by the cells into the medium. The significance of these factors has been discussed previously (Powell, 1957). Apart from the medium lacking embryo extract, increased frequencies of damaged cells were associated with high proliferation rates and resulting exhaustion of media (No. 3 and 6).

The effects of density of population were further investigated by varying the proportions of ascitic fluid to ascitic plasma but maintaining the combined total of these at 37½ per cent of the complete basic medium. The results of a typical experiment are given in Table III and illustrate the characteristic relation between density of population and cell multiplication and degeneration, respectively. The incidence of eosin-stained cells was also related to the increase in population during the incubation period.

TABLE III.—*Effects of Increasing Initial Cell Populations upon Proliferation in Basic Medium*

The two higher initial populations were calculated on the basis of that determined for the lowest.

| Initial population | Population after 24 hours (mm.$^3$) | Percentage difference in population after 24 hours | Percentage of eosin-stained cells after 24 hours |
|---|---|---|---|
| 17,614 | 23,850 | +35·9 | 2·9 |
| 35,228 | 35,000 | ± | 1·8 |
| 52,842 | 47,350 | −10·3 | 24·4 |

Initial population densities significantly less than 1/8th of the *in vivo* density were found to be unfavourable for rapid multiplication. This was attributed to the inability of the cells in these numbers to produce an adequate concentration of diffusible protective factors in the medium. In general it appeared that in 2·5 ml. of the basic medium a tumour cell population of about 1/8th of the normal range in native ascitic fluid gave a convenient compromise between cell proliferation and degeneration during an incubation period of 24 hours.

Attempts were made to cultivate Sarcoma 37 ascites tumour cells in similar basic medium with homologous ascitic plasma. These attempts failed. However, multiplication rates comparable to those found *in vivo* were obtained with a modified medium in which ascitic fluid constituted 1/8th of the total volume. In this one of the two parts of the embryo extract was replaced with one of tumour cell extract; in all other respects the basic medium was unchanged. To prepare this extract the sedimented cells of centrifuged Sarcoma 37 ascitic fluid were suspended in an amount of Earle's solution equal in volume to that of the supernatant plasma removed and the suspension incubated for 3 hours at 37°. The cell suspension was shaken at intervals during the incubation, finally centrifuged and the supernatant used as extract.

The tumour cell extract presumably contained ample essential solutes not sufficiently provided in the unmodified basic medium. The practical limitation of the latter set by the necessity of using a relatively high initial population of tumour cells to obtain adequate multiplication and the consequent depletion of the medium within 24 hours was overcome by the inclusion of the tumour cell extract in the basic medium. Sarcoma 37 tumour cells grew in this supplemented medium for several days when initial population densities 1/100th of that of the native ascitic fluid were used. Ehrlich carcinoma ascites tumour cells behaved similarly in supplemented homologous media in which the soluble factors were supplied by the cell extract.

### DISCUSSION

The media described were able to support the growth *in vitro* of the ascites tumour cells tested. They had the disadvantage that two of the components were prepared from native ascites tumour fluid. No suitable substitutes for ascitic plasma and serum or tumour cell extract were found. The latter was not entirely replaceable by embryo extract.

Ehrlich carcinoma and Sarcoma 37 ascites tumour cells differed quantitatively in their dependence on the concentration in the culture medium of the soluble essential substances released by the cells and supplied in the cell extract. The ascitic plasma used presumably contained a low proportion of these substances since Ehrlich carcinoma cells grew at certain population densities without the addition of cell extract. The importance of these soluble factors for the viability and growth of ascites tumour cells has been discussed previously (Powell, 1957). The present researches confirmed this earlier work. The limiting factors in the dependence of the tumour cells upon these substances may be the ratios between the rates at which they are synthesised and exchanged between the cells and medium. The tumour cell extract component of the media may be more important for the growth of the tumour cells than the plasma fraction.

The media described would appear to be suitable for quantitative studies on the effects of cytotoxic agents on tumour cell populations under controlled conditions *in vitro*. Relatively large initial populations of tumour cells, 1/8th of the *in vivo* density, could be studied for periods of 24 hours or smaller initial populations for longer periods. Cultivated tumour cells inoculated intra-peritoneally into mice gave rise to normal ascites tumours.

The roles of the enzymes used in the basic medium are uncertain. Trypsin has been used frequently to dissociate the cells of tissues; Moscona (1952), and Moscona and Moscona (1952) used it upon chick embryo tissues. Willmer (1945, 1954) has described its use for this purpose and also its effect in causing fibrocytes to round up with retention of viability. This latter property may be associated with its beneficial effects in the medium. On the other hand, it is improbable that the enzyme remained active for long periods in the presence of the considerable amounts of protein in the medium. It is possible that its proteolytic effect upon the ascitic plasma was important since carcinoma cells liberate proteolytic enzymes and the ascites cells were cultivated at densities of population lower than those *in vivo*. The enzyme perhaps compensated for the reduced numbers of cells in this respect and may have liberated nutrients form the plasma proteins.

Hyaluronidase, which depolymerises hyaluronic acid derivatives, reduces cartilaginous matrix *in vitro* (Paff and Seifter, 1950) and dissolves intercellular cementing substance. Possibly it affected the surfaces of the ascites cells. Embryo extract presumably functioned in virtue of its content of growth substances.

During the past decade many reports of successful cultivation of cell suspended in fluid media have been made (Paul, 1959). These include L strain fibroblasts (Earle *et al.*, 1954, 1956 ; Danes, 1957 ; McLimans *et al.*, 1957), HeLa human carcinoma cells (Gey, Bang and Gey, 1954; Earle *et al.*, 1956). In most of these instances the suspended cells have been subjected to violent continuous agitation and standard media have been used. A substrain of de Bruyn's MB lymphoblast has been grown in slow suspension by Owens, Gey and Gey (1953) but these cells are atypical and altered from the parent strain.

The present researches differ from these examples of true suspension cultures in that the cells lay mainly on the surfaces of the culture vessels although smoothly contoured dividing cells were found free in the medium and suspensions of cells were easily recovered from the roller-tubes. As with the lymphoblast cells of de Bruyn the present results were probably due to the innate properties of the ascites tumour cells reinforced by a medium consisting largely of their normal pabulum.

### SUMMARY

A fluid culture medium which supports the growth of Ehrlich carcinoma ascites tumour cells *in vitro* is described.

This medium is suitable for short term assays of the effects of cytotoxic agents on the cultivated tumour cells, since they can be quantitatively recovered for enumeration.

The numbers of cells inoculated into this medium must lie within narrow limits for successful cultivation.

Supplementation of the basic medium with a saline extract of homologous ascites tumour cells permits successful cultivation with much smaller initial numbers of cells. Sarcoma 37 ascites tumour cells, which fail to grow in the basic medium, multiply in supplemented medium.

This saline extract is presumed to contain soluble protective factors which diffuse from the cells into the medium. The concentration of these substances determines the number of cells which may be successfully inoculated in cultures.

I am indebted for assistance with the *in vitro* researches to Mr. G. A. Butcher and with the maintenance of the tumours *in vivo* to Mr. F. Butcher.

The expenses of this work were defrayed from a block grant by the British Empire Cancer Campaign.

### REFERENCES

British Empire Cancer Campaign.—(1951) *Ann. Rep.*, **29**, 57.

Danes, B. S.—(1957) *Exp. Cell. Res.*, **12**, 169.

Earle, W. R.—(1943) *J. nat. Cancer Inst.*, **4**, 165.

*Idem*, Bryant, J. C., Schilling, E. L. and Evans, V. J.—(1956) *Ann. N.Y. Acad. Sci.*, **63**, 666.

*Idem*, Schilling, E. L., Bryant, J. C. and Evans, V. J.—(1954) *J. nat. Cancer Inst.*, **14**, 1159.

Gey, G. O., Bang, F. B. and Gey, M. K.—(1954) *Tex. Rep. Biol. Med.*, **12**, 805.

Heilbrunn, L. Y. and Wilson, W. L.—(1949) *Proc. Soc. exp. Biol., N.Y.*, **70**, 179.

Klein, G.—(1950) *Cancer*, **3**, 1052.—(1951) 'The Production of Ascites Tumours in Mice and Their Use in Studies on Some Biological and Chemical Characteristics of Neoplastic Cells'. Uppsala (Almqvist and Wicksells Botryckeri).

*Idem* and Révész, L.—(1953) *J. nat. Cancer Inst.*, **14**, 229.

Lettré, H.—(1941) *Z. physiol. Chem.*, **268**, 59 ; **271**, 192.—(1950) *Z. Krebsforsch.*, **57**, 1.

McLimans, W. F., Giardinello, F. E., Davis, E. V., Kucera, C. J. and Rake, G. W. —(1957) *J. Bact.*, **74**, 768.

Moscona, A.—(1952) *Exp. Cell Res.* **3**, 535.

Moscona, H. and Moscona, A.—(1952) *J. Anat.*, **86**, 287.

Owens, O. von H., Gey, G. O. and Gey, M. K.—(1953) *Proc. Amer. Ass. Cancer Res.*, **1**, 41.

Paff, G. H. and Seifter, J.—(1950) *Anat. Rec.*, **106**, 525.

Paul, J.—(1959) 'Cell and Tissue Culture'. London (Livingston).

Powell, A. K.—(1957) *Brit. J. Cancer*, **11**, 274, 280, 478.

Schrek, R.—(1936) *Amer. J. Cancer*, **28**, 389.

Warburg, O. and Heipler, E.—(1952) *Z. Naturf.*, **7b**, 193.

Willmer, E. N.—(1945) 'Growth and Form in Tissue Cultures'. *In* 'Growth and Form Essays presented to D'Arcy Thompson', ed. W. Le Gros Clark and P. B. Medawar. Oxford (University Press).—(1954) 'Tissue Culture', 2nd edition. London (Methuen).